# Participation in lung cancer screening programs: are there gender and social differences? A systematic review

**DOI:** 10.1186/s40985-018-0100-0

**Published:** 2018-08-15

**Authors:** Stefanie Schütte, Damien Dietrich, Xavier Montet, Antoine Flahault

**Affiliations:** 1 Centre Virchow-Villermé for Public Health Paris-Berlin, Université Sorbonne Paris Cité, Paris, France; 2 Institute of Global Health, Faculty of Medicine, University of Geneva, 9, chemin des mines, 1202 Geneva, Switzerland; 3 Radiology and Medical Informatics Department, Geneva University Hospitals, Geneva, Switzerland

**Keywords:** Lung cancer, Screening, Gender, Socio-economic determinants, Review

## Abstract

Lung cancer remains the leading cause of cancer mortality worldwide. A number of screening trials for early detection of lung cancer exist, using chest X-ray, low-dose computed tomography, or both. However, little is known about the socio-demographic characteristics of participants in lung cancer screening programs. As gender and socio-economic determinants are important variables to consider for successful program implementation, this review aims to characterize the participants in such programs and to investigate whether differences in representation exist across screening programs.

Systematic methods were used to identify relevant studies. A search was undertaken to locate all studies published up to August 2017 assessing the socio-demographic profile of participants in lung cancer screening programs. A search strategy was developed, refined, and implemented to search in two different online databases (MEDLINE and Web of Sciences).

A total of 1588 references were retrieved of which 14 were eligible for review. The results highlight differences in gender and social characteristics of participants across programs, while noting that differences may be partly explained by the various epidemiological contexts, program inclusion criteria, and socio-economic status (SES) measures collected. Most importantly, despite a well-recognized predominance of low SES among heavy smokers, people with high SES are seemingly over-represented among participants. Male participants also seem to be over-represented. These findings are important to help inform the development and implementation processes of future lung cancer screening programs, which should likely include strategies for engaging women as well as individuals with low SES and, of course, those most at risk for developing lung cancer.

## Background

Lung cancer remains the leading cause of cancer mortality worldwide [1]. The overall 1-year survival rate for small cell lung cancer has remained between 12 and 16% over the last decades [2]. Although lung cancer mortality is decreasing in some regions, globally, mortality from the disease continues to rise. Prevention and smoking cessation are still the main methods to reduce mortality due to lung cancer. Despite major achievements, research indicates that lung cancer will remain a major cause of death worldwide for several decades [3].

Since disease stage at the time of diagnosis is the most important determinant of prognosis, efforts are being made to increase early detection of lung cancer, allowing early commencement of suitable therapy and improved prognosis.

A number of screening trials for early detection of lung cancer have been, or are being, conducted using both chest X-ray (CXR) and low-dose computed tomography (LDCT) screening. The latter is the superior method; it is more sensitive than CXR and has enabled detection of small, asymptomatic lung tumors. Early diagnosis by LDCT screening led to a substantial 20% reduction in lung cancer-specific mortality and a significant 6.7% reduction in all-cause mortality in the US National Lung Screening Trial [4]. A lung cancer screening program with LDCT is a complex endeavor with the purpose of identifying asymptomatic patients affected by lung cancer at an early stage, thereby maximizing the odds of a curative treatment without causing harm to healthy participants. As LDCT screening is currently being implemented on an extensive, population-wide, scale in several countries [5, 6], it seems probable that lung cancer screening will enter the health care arena, irrespective of whether it is privately or publically funded. However, several expert panels have highlighted the need for further improvements of LDCT screening before implementation [7].

Compared to routine screening for other types of cancers, screening for lung cancer relies mainly on age and requires consideration of smoking status intensity. Currently, the United States Preventive Services Task Force recommends (grade B) annual LDCT screening for individuals aged between 55 and 80 years having a minimum smoking history of 30 pack-years, who either currently smoke or have quit within the past 15 years, and who do not have a prior malignancy [8].

Successful implementation of lung cancer screening depends on being able to reach high-risk individuals. However, research has shown that those at higher risk are less interested in being screened despite recognizing that they are at risk [9]. Moreover, little is known about the socio-demographic characteristics of participants in lung cancer screening. The incidence and outcome rates of lung cancer show social differences, with incidence and mortality rates 2 to 3 times higher in lower socio-economic groups [10]. It has previously been shown that individuals of lower socio-economic status (SES), smokers, and younger individuals are less likely to take part to the first stage of recruitment to the UKLS trial [11]. Indeed, reduced uptake of participants for cancer screening has been consistently observed among deprived populations [12] and is also associated with factors such as marital status, health insurance coverage status, type of residential area, and ethnicity [13, 14]. Individuals with lower SES may face greater barriers with regard to both logistics (e.g., travel, childcare responsibilities, inflexible work-hours) and communication (e.g., language, literacy), rendering it more difficult for them to adhere and to attend cancer screening programs. Previous studies have also highlighted some of the barriers and facilitators to lung cancer screening [15, 16].

Gender and socio-economic determinants are thus important variables to consider for the successful implementation of cancer screening programs. Therefore, this review aims to characterize the representation of participants in such programs and to investigate whether gender and social differences exist across programs.

## Methods

Systematic methods were used to identify relevant studies and to assess their eligibility for inclusion. The review was reported according to the Preferred Reporting Items for Systematic Reviews and Meta-Analyses (PRISMA) guidelines [17].

A search was undertaken to locate all studies published up to August 2017 assessing the socio-demographic profile of participants in lung cancer screening. A search strategy was developed, refined, and used to search in two different online databases (MEDLINE and Web of Sciences). The search terms were adapted according to the database (for example, MEDLINE recognizes the MESH term Lung Neoplasms whereas Web of Science does not). EndNote software was used to manage references. Keywords and terms used for the search included the following: lung cancer, lung carcinoma, lung neoplasms; screening, detection; social class, education, income, deprivation, socio-demographic, gender and sex. Table 1 shows the detailed search strategy for each database.

**Table 1:** Characteristics of included studies

	*N* (total = 14)
Type of lung cancer screening	
CXR	3
LDCT	7
Both	4
Measure of social status	
Education	6
Family/household income	2
Occupation	2
Deprivation	1
None	5
Other variables	
Age	10
Race/ethnicity	4
Marital status	4
Smoking status	9
Insurance status	1
Employment status	1
None	3
Geographical focus	
Asia	3
Europe	7
North America	4

The search was limited to articles published in English. Study selection was performed in two stages. Firstly, abstracts were examined using the following inclusion and exclusion criteria: original research (quantitative studies) with a focus on lung cancer screening including socio-demographic information of participants (gender and/or SES) as descriptive variable or confounder published until August 2017. Exclusion criteria were as follows: no original research (e.g., letters), editorials, reviews, and qualitative studies.

Secondly, titles and abstracts of identified studies were screened for relevance to the topic. Those studies considered not to be relevant on the basis of topic were excluded. The selected full papers were then assessed for eligibility according to the study eligibility criteria. Disagreements at any of the screening stages were resolved by discussion between reviewers. Full manuscripts were obtained for all publications included after the first evaluation. As a further step, we excluded studies that had a focus on minorities or for which data were not directly extractable. In case of multiple publications relating to the same study, the latest reference in which relevant data were reported was considered.

**Fig. 1:**
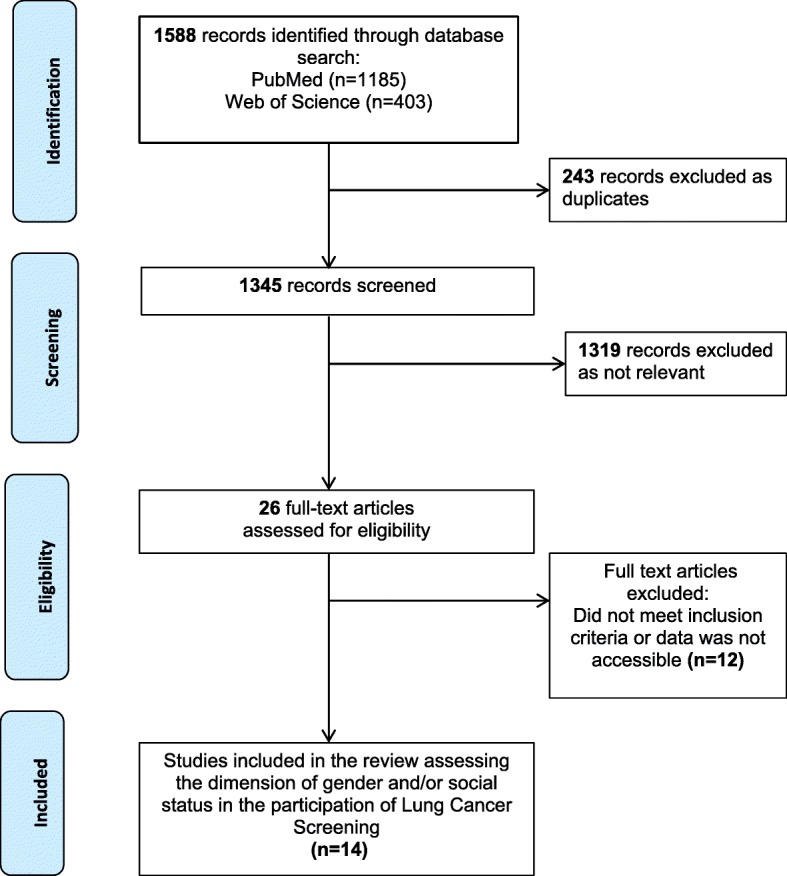
Process of study selection

Identified studies were assessed and reported using the PRISMA flow diagram (Fig. 1). Data relating to study authors, year published, journal, type of screening, number of participants, data source, population included, gender, measure of social status, and other variables were retrieved. Prevalence rates for the variables gender and SES were extracted for each study sample. In most cases, the highest two categories of the SES variable were grouped together in order to estimate the percentage of participants having high SES. For instance, one publication [18] classified the participants in four educational categories: (1) less than high school graduate, (2) high school graduate/GED holder, (3) some college/associate degree, and (4) bachelor’s degree and more. In this case, educational categories 3 and 4 were grouped together to obtain a “high SES” category. This procedure allows comparison of SES across studies despite the fact that the SES indicators used vary.

In the field of systematic reviews, scores are often allocated to reflect desirable features related to the validity of the study. As the designs of included studies were quite heterogeneous, no quality scoring was applied to this review.

## Results

Figure 1 presents a flow diagram outlining the systematic review process. A total of 1588 references were retrieved by the searches of MEDLINE and Web of Science, of which 14 were eligible for this review. Table 1 shows the characteristics of included studies. Half (*n* = 7) of the selected studies used LDCT as the screening method, three out of the 14 studies used CXR, and four other studies included both modalities. Except for one study [19], all studies conducted stratified analyses by gender. Six studies reported education as a measure of SES. Family/household income, occupation, or an index of deprivation were also occasionally reported. Of note, five studies did not use an indicator of SES. Most of the selected studies used age and smoking status as co-variables. Variables such as race/ethnicity and marital status were also found in four studies. In terms of geographical focus, three studies came from Asia, seven from Europe, and four from North America.

Table 2 shows the summary of details of the reviewed publications. Overall, the number of participants ranged from 378 in Japan to 53,456 in the USA. The first two articles on this topic were published in 2002. Both studies were conducted in Japan. One was a Japanese case-control study and CXR was the modality used [20]. The other article consisted of participants from Hitachi Employee’s Health Insurance Group and LDCT was used [21]. Four studies were conducted in the USA, among them was the large National Lung Screening Trial (NLST), comparing both screening methods [22]. Doria-Rose and colleagues conducted a National Health Interview Survey among participants in the USA using either type of screening [18]. In 2003, another US study examined data of 4705 participants in CXR screening, which were part of the large Prostate, Lung, Colorectal and Ovarian Cancer Screening (PLCO) [23].

**Table 2:** Summary of details of the reviewed publications

Year of publication	First author	Type of screening	Number of participants	Study design	Measure of social status	Other variables
2002	Nakayama et al.	CXR	536 Japan	Case-control study	No	Age, smoking status
2002	Nawa et al.	LDCT	7956 Japan	Cohort study	No	No
2003	Ford et al.	CXR	4705 USA	Randomized controlled trial	Education	Race, age, smoking status
2006	Kamposioras et al.	CXR	1099 Greece	Cross-sectional study	Occupation	Age, smoking status, family history of lung cancer
2007	Blanchon et al.	LDCT CXR	765 France	Randomized controlled trial	No	Age, smoking status
2009	Pegna et al.	LDCT	3206 Italy	Randomized controlled trial	No	No
2010	Aberele et al.	LDCT CXR	53,456 USA	Randomized controlled trial	Education	Race, age, ethnicity, marital status, weight, smoking status
2010	Van der Aalst et al.	LDCT	5161 The Netherlands, Belgium	Randomized controlled trial	Education	Age, marital status
2011	Hestbech et al.	LDCT	4101 Denmark	Cross-sectional study	Education, Occupation	Geographical area, living alone, smoking status, employment status
2011	Kondo et al.	LDCT CXR	378 Japan	Retrospective study	No	No
2011	Wildstein et al.	LDCT	3387 USA	Cohort study	Education	Age, ethnicity, family history
2012	Doria-Rose et al.	LDCT CXR	619 USA	Cross-sectional study	Education, family income	Race, age, insurance status, unemployment, smoking status
2014	McRonald et al.	LDCT	23,794 UK	Randomized controlled trial	Index of multiple deprivation (IMD)	Age, smoking status
2014	Zakrzewska et al.	LDCT	1619 Poland	Cross-sectional study	Income per household	Age

The LDCT screening method was also assessed among participants in the framework of the Early Lung Cancer Action Program in the USA [24]. In addition, one large study from the UK of a LDCT screening was included in this review [11]. Several studies from various European countries with a focus on LDCT screening were also included in this review: Denmark using data from the Danish Lung Cancer Screening Trial including 4101 participants [25], France with 765 participants [26], Italy with ITALUNG and its 3206 participants [27], the Netherlands and Belgium with 5161 participants [19], and one study from Poland with 1619 participants [28]. Except for the French and Italian studies, all of the included European studies used a measurement of social status. Moreover, a Greek study that assessed lung cancer screening through CXR used occupation as a social indicator [29].

The inclusion criteria of these studies were very different. Nakayama et al. included high-risk males and non-high-risk females. The definition of high-risk was 600 or more on the smoking index (average number of cigarettes smoked per day multiplied by the number of years of regular smoking) [20]. Other studies included current and past smokers as defined “[someone] who smokes or smoked cigarettes regularly” [23] or participants were categorized according to the number of daily cigarettes smoked (< 10, 10–20, 20–30, 30–40, > 40, no smokers) [29].

In the French study, individuals were selected with a current or former cigarette smoking history of ≥ 15 cigarettes per day for at least 20 years (former smokers having quit < 15 years prior to enrollment) [26]. The requirement was slightly higher for the Italian and Danish study (cigarette smoking history of ≥ 20 cigarettes per day) [25, 27] as well as for the US national trial (≥ 30 cigarettes per day) [22]. Participants in the NELSON trial were included with a smoking history of > 15 cigarettes a day for > 25 years or > 10 cigarettes a day for > 30 years, current smoking, or former smokers who quit smoking < 10 years ago [19]. Wildstein and colleagues described individuals participating in their study as 40 years of age or older with a smoking history of at least one pack-year, no prior cancer, and no CT in the prior 3 years [24]. The cross-sectional study by Doria-Rose et al. divided participants into two categories: “higher risk” smokers were those with a 30 pack-year or more smoking history and were either current smokers or former smokers who had quit within 15 years prior to the interview, while “lower risk” smokers had either a fewer than 30 pack-year history and/or had quit more than 15 years ago [18]. Eight studies had also information on smoking status (never/former/current smoker), and among these studies, a high proportion of participants were never or former smokers.

Table 3 presents the reported social and gender characteristics of the participants in lung cancer screening programs. The main focus of the selected articles was either for clinical purposes or for presenting the baseline characteristics of study participants. The majority of included articles showed the characteristics of study participants using univariate analyses but in multivariate analyses, no study stratified data by gender. Only one study [18] gave the percentage of female or male participants by adjusting for potential confounders. Therefore, the results have to be considered with caution.

**Table 3:** Social and gender characteristics of participants to lung cancer screening programs

First author	Gender (% of men among participants)	Socio-economic status (% of high SES among participants)
Aberle et al. 2010	59.0	54.7 (education)
Blanchon et al. 2007	70.5	
Doria-Rose et al. 2012	57.6	47.4 (education)
Ford et al. 2003	45.0	69.3 (education)
Hestbech et al. 2011	55.3	47.2 (education)
Kamposioras et al. 2006	46.4	31.1 (occupation)
Kondo et al. 2011	22.0	
McRonald et al. 2014	50.0	57.9 (deprivation index)
Nakayama et al. 2002	74.6	
Nawa et al. 2002	79.4	
Pegna et al. 2009	64.7	
Van der Aalst et al. 2010	Only males	52.0 (education)
Wildstein et al. 2011	47.0	60.0 (education)
Zakrzewska et al. 2014	51.0	44.3 (Income)
Mean	55.6	51.5

Overall, men were slightly more likely to participate in lung cancer screening programs, although the gender ratio varied across studies from 22 to 79%. The publication of Kondo et al. was the only study that reported a very low percentage of male participants in a Japanese cohort study [30]. This was also the study with the lowest number of participants (*n* = 378). The highest male participation was found in another Japanese study exploring LDCT screening [21]. No significant gender differences were found according to type of screening (LDCT and CXR). It should be noted that the inclusion criteria varied across the selected studies and we cannot rule out a possible impact on our results.

The results for SES also varied across studies with the percentage of participants with high SES ranging from 31 to 69%. One study showed a 31% prevalence of high SES among participants [29], but for all remaining studies, this percentage was above 40%. Only two separate studies focusing on CXR included SES indicators in their analyses. The first reported 69% [23] of participants as having high SES and the other 31% [29].

## Discussion

Strategies to ensure equitable participation are critical for the successful implementation of new cancer screening programs. While designing a preventive program, it is crucial to know the target group and the main factors that induce people to participate.

Out of 1588 studies, 14 were found to be relevant for this review, presenting the gender or social characteristics of participants in lung cancer screening programs. This review highlighted differences in the gender and social characteristics of the participants across the different programs, although these differences may be partly explained by the different epidemiological contexts, program inclusion criteria, and measures collected. Most importantly, despite a well-recognized predominance of low SES among heavy smokers, people with a high SES are seemingly over-represented among participants. This could be definitely ascertained by a direct comparison of adjusted SES between participants and non-participants coming from the same population. However, these data were not, to our knowledge, available for most of the studies, except for the Danish Study [25] which is further described below. Interestingly, the percentage of men among participants greatly varied across studies. Overall, these results raise concern about access to lung cancer screening programs for people with a low SES.

A slightly higher percentage of male participants has been observed in other screening studies [31] and may be due to the fact that women are more accustomed to breast and cervical cancer screening programs. Moreover, this percentage varied across studies and must be interpreted with extra caution when not adjusted for smoking status. Indeed, men usually have a higher prevalence of smoking than women [32].

The socio-economic and personal data were self-reported in most of the selected studies and may bias the results, especially when it comes to financial questions such as monthly (household/family) income.

There are several factors that may influence the link between SES and screening participation. Low SES is likely to be associated with later stage at diagnosis for many cancer types and also with a greater prevalence or severity of comorbid conditions, which may prevent participation. Previous studies have shown that low participation among individuals with low SES relates to poorer self-reported health [33].

Importantly, the Danish study from Hestbech et al. showed that participants had higher SES and reported less negative psychosocial aspects compared to ordinary heavy smokers from the general population. This tends to confirm that people with a high SES seem over-represented among participants in lung cancer screening programs. Also, more men participated, which is in agreement with the known gender ratio among Danish heavy smokers [25].

Almost all included studies in this review showed a high percentage of former or never smokers. This may be explained by the fact that current smokers are more likely to report emotional barriers as reasons for non-participation [34]. As individuals from higher SES are more likely to quit smoking [35], the apparent social inequalities may be underestimated because of the high proportion of former smokers across the studies.

Barriers that limit participation in screening programs are complex and multifactorial. It has been reported that other factors such as difficulties with traveling to attend screening, comorbidity, and career responsibilities were the most common self-reported reasons for non-participation in screening programs. Other studies found that cost of the LDCT was a barrier to willingness to be scanned [36]. Wildstein and colleagues suggested that removing financial barriers by offering free screenings or health insurance coverage might facilitate participation in a lung cancer screening program [24].

There were a number of limitations to this review. First, we considered only the crude percentage of male and female participation and could not take into account the weights of sample sizes. Second, though a pool was used to allow comparison between studies, some results were discordant at the level of the initial SES indicator examined. Choosing a measure of SES is a complex task because the appropriateness of a measure depends on the social context and may differ across countries, cultures, and time [37]. As this review included studies from various countries with different cultural and health care services that were conducted in different settings and years, there was heterogeneity in the measurements of SES indicators, which may have an impact of the results of this review. Moreover, because these measures varied considerably across studies, we could not compare the quality of included studies nor perform a formal meta-analysis. Third, the recruitment of participants also varied across studies, explaining part of the differences observed. The participants of the Dutch–Belgian Lung cancer Screening Trial (NELSON) were recruited by the use of population registries with the aim to eliminate the risk of selection bias. Participants in the trial ITALUNG were enrolled from lists of residents in the screening center areas registered with their physician [27], whereas for most other studies, enrolled individuals presented voluntarily to screening centers asking for lung cancer screening. In addition, Aberle and colleagues stated that study participants are often healthier as well as better educated than those to whom study results are to be generalized [22]. Fourth, prevalence rates of participation and the modalities of participation may vary over time and according to the country. This could explain some of the variation between the identified studies. For instance, the morbidity and mortality rates due to lung cancer differ significantly between countries, with Japan presenting very high rates. Moreover, lung cancer screening is fairly widespread for not only smokers but also for never-smokers in East Asia and particularly in Japan [30], which is the opposite of what is recommended in Europe or in the USA. The selected studies all came from high-income countries, and results are therefore not valid for low- and middle-income countries. Literature has also shown that the participation in lung cancer screening programs varies according to ethnicity. For instance, African Americans were more likely than whites to avoid lung cancer screening [38]. Fifth, the restriction to English-language articles excluded studies published in other languages. However, we looked at studies that were excluded based on the language (66 articles) to confirm that these papers did not meet our other selection criteria. Sixth, we also excluded non-published studies and reports. Publication bias can result in significant associations being preferentially published, which might be an even greater issue for secondary exploratory analyses of factors or subgroups, such as included in this review.

Despite its limitations, this is the first systematic review of the literature on the social determinants of participation in lung cancer screening that includes both screening methods: CXR and LDCT. We believe that our emphasis on gender and social differences can be influential for a wider range of studies looking at the participation in lung cancer screening programs.

## Conclusion

Lung cancer screening programs were developed in order to decrease the overall mortality in the screened population and the lung cancer-specific mortality, which has been shown in various studies. However, lung cancer screening with LDCT is a complex and controversial topic and has inherent risks and benefits. This screening method has a high false-positive rate, and this may result in unnecessary harm, such as anxiety or inappropriate interventions, which should be taken into account before implementation.

This review identified existing gender and social differences in the participation in various screening programs, although these differences may be partly explained by the different epidemiological contexts, program inclusion criteria, and SES measures collected. Nevertheless, participants in lung cancer screening programs seem to have higher SES compared to the general source population. Male participants are also seemingly over-represented. These findings are important to help inform the development and implementation processes of future lung cancer screening programs, which should likely include strategies for engaging women as well as individuals with low SES and, of course, those most at risk. To ensure broad participation in lung cancer screening, knowledge of attitudes and beliefs about screening across ethnic groups as well as barriers relating to access should also be considered in the future.

## References

[1] Lozano R, Naghavi M, Foreman K, Lim S, Shibuya K, Aboyans V (2012). Global and regional mortality from 235 causes of death for 20 age groups in 1990 and 2010: a systematic analysis for the Global Burden of Disease Study 2010. Lancet Lond Engl.

[2] Walters S, Maringe C, Coleman MP, Peake MD, Butler J, Young N (2013). Lung cancer survival and stage at diagnosis in Australia, Canada, Denmark, Norway, Sweden and the UK: a population-based study, 2004–2007. Thorax.

[3] Islami F, Torre LA, Jemal A (2015). Global trends of lung cancer mortality and smoking prevalence. Transl Lung Cancer Res.

[4] Aberle DR, Adams AM, Berg CD, Black WC, Clapp JD, National Lung Screening Trial Research Team (2011). Reduced lung-cancer mortality with low-dose computed tomographic screening. N Engl J Med.

[5] Wood DE (2015). National Comprehensive Cancer Network (NCCN) clinical practice guidelines for lung cancer screening. Thorac Surg Clin.

[6] Zhou Q-H, Fan Y-G, Bu H, Wang Y, Wu N, Huang Y-C (2015). China national lung cancer screening guideline with low-dose computed tomography (2015 version). Thorac Cancer.

[7] Frauenfelder T, Puhan MA, Lazor R, von Garnier C, Bremerich J, Niemann T (2014). Early detection of lung cancer: a statement from an expert panel of the Swiss university hospitals on lung cancer screening. Respir Int Rev Thorac Dis.

[8] Moyer VA, U.S. Preventive Services Task Force. Screening for lung cancer: U.S. Preventive Services Task Force recommendation statement. Ann Intern Med 2014;160(5):330–338. 10.7326/M13-2771 24378917

[9] Silvestri GA, Nietert PJ, Zoller J, Carter C, Bradford D (2007). Attitudes towards screening for lung cancer among smokers and their non-smoking counterparts. Thorax.

[10] Sidorchuk A, Agardh EE, Aremu O, Hallqvist J, Allebeck P, Moradi T (2009). Socioeconomic differences in lung cancer incidence: a systematic review and meta-analysis. Cancer Causes Control CCC.

[11] McRonald FE, Yadegarfar G, Baldwin DR, Devaraj A, Brain KE, Eisen T (2014). The UK Lung Screen (UKLS): demographic profile of first 88,897 approaches provides recommendations for population screening. Cancer Prev Res Phila Pa.

[12] Szczepura A, Price C, Gumber A (2008). Breast and bowel cancer screening uptake patterns over 15 years for UK south Asian ethnic minority populations, corrected for differences in socio-demographic characteristics. BMC Public Health.

[13] Hsia J, Kemper E, Kiefe C, Zapka J, Sofaer S, Pettinger M (2000). The importance of health insurance as a determinant of cancer screening: evidence from the Women’s Health Initiative. Prev Med.

[14] Lorant V, Boland B, Humblet P, Deliège D (2002). Equity in prevention and health care. J Epidemiol Community Health.

[15] van den Bergh KAM, Essink-Bot ML, van Klaveren RJ, de Koning HJ (2009). Informed participation in a randomised controlled trial of computed tomography screening for lung cancer. Eur Respir J.

[16] Patel D, Akporobaro A, Chinyanganya N, Hackshaw A, Seale C, Spiro SG (2012). Attitudes to participation in a lung cancer screening trial: a qualitative study. Thorax.

[17] Moher D, Liberati A, Tetzlaff J, Altman DG (2009). Preferred reporting items for systematic reviews and meta-analyses: the PRISMA statement. Ann Intern Med.

[18] Doria-Rose VP, White MC, Klabunde CN, Nadel MR, Richards TB, McNeel TS (2012). Use of lung cancer screening tests in the United States: results from the 2010 National Health Interview Survey. Cancer Epidemiol Biomark Prev Publ Am Assoc Cancer Res Cosponsored Am Soc Prev Oncol..

[19] van der Aalst CM, van den Bergh KAM, Willemsen MC, de Koning HJ, van Klaveren RJ (2010). Lung cancer screening and smoking abstinence: 2 year follow-up data from the Dutch-Belgian randomised controlled lung cancer screening trial. Thorax.

[20] Nakayama T, Baba T, Suzuki T, Sagawa M, Kaneko M (2002). An evaluation of chest X-ray screening for lung cancer in Gunma prefecture, Japan: a population-based case-control study. Eur J Cancer Oxf Engl 1990.

[21] Nawa T, Nakagawa T, Kusano S, Kawasaki Y, Sugawara Y, Nakata H (2002). Lung cancer screening using low-dose spiral CT: results of baseline and 1-year follow-up studies. Chest.

[22] Aberle DR, Berg CD, Clapp JD, Clingan KL, Gareen IF, Lynch DA (2010). Baseline characteristics of participants in the randomized national lung screening trial. J Natl Cancer Inst.

[23] Ford ME, Havstad SL, Flickinger L, Johnson CC. Examining the effects of false positive lung cancer screening results on subsequent lung cancer screening adherence. Cancer Epidemiol biomark Prev Publ am Assoc Cancer res cosponsored am Soc Prev Oncologia 2003 12(1):28–33. 12540500

[24] Wildstein KA, Faustini Y, Yip R, Henschke CI, Ostroff JS (2011). Longitudinal predictors of adherence to annual follow-up in a lung cancer screening programme. J Med Screen.

[25] Hestbech MS, Siersma V, Dirksen A, Pedersen JH, Brodersen J (2011). Participation bias in a randomised trial of screening for lung cancer. Lung Cancer Amst Neth.

[26] Blanchon T, Bréchot J-M, Grenier PA, Ferretti GR, Lemarié E, Milleron B, et al. Baseline results of the Depiscan study: a French randomized pilot trial of lung cancer screening comparing low dose CT scan (LDCT) and chest X-ray (CXR). Lung Cancer Amst Neth. 2007;58(1):50–8. 10.1016/j.lungcan.2007.05.009 17624475

[27] Lopes Pegna A, Picozzi G, Mascalchi M, Maria Carozzi F, Carrozzi L, Comin C (2009). Design, recruitment and baseline results of the ITALUNG trial for lung cancer screening with low-dose CT. Lung Cancer Amst Neth..

[28] Zakrzewska A, Szczepanowska M, Książek J, Biadacz I, Dziedzic R, Jelitto-Górska M (2014). The influence of selected factors on the attendance of the high-risk population in the early lung cancer detection program. Pneumonol Alergol Pol.

[29] Kamposioras K, Casazza G, Mauri D, Lakiotis V, Cortinovis I, Xilomenos A (2006). Screening chest radiography: results from a Greek cross-sectional survey. BMC Public Health.

[30] Kondo R, Yoshida K, Kawakami S, Shiina T, Kurai M, Takasuna K (2011). Efficacy of CT screening for lung cancer in never-smokers: analysis of Japanese cases detected using a low-dose CT screen. Lung Cancer Amst Neth..

[31] von Euler-Chelpin M, Brasso K, Lynge E (2010). Determinants of participation in colorectal cancer screening with faecal occult blood testing. J Public Health Oxf Engl.

[32] Bolego C, Poli A, Paoletti R (2002). Smoking and gender. Cardiovasc Res.

[33] Miles A, Rainbow S, von Wagner C (2011). Cancer fatalism and poor self-rated health mediate the association between socioeconomic status and uptake of colorectal cancer screening in England. Cancer Epidemiol Biomark Prev Publ Am Assoc Cancer Res Cosponsored Am Soc Prev Oncol.

[34] Ali N, Lifford KJ, Carter B, McRonald F, Yadegarfar G, Baldwin DR (2015). Barriers to uptake among high-risk individuals declining participation in lung cancer screening: a mixed methods analysis of the UK Lung Cancer Screening (UKLS) trial. BMJ Open.

[35] Albertsen K, Borg V, Oldenburg B (2006). A systematic review of the impact of work environment on smoking cessation, relapse and amount smoked. Prev Med.

[36] Jonnalagadda S, Bergamo C, Lin JJ, Lurslurchachai L, Diefenbach M, Smith C (2012). Beliefs and attitudes about lung cancer screening among smokers. Lung Cancer Amst Neth..

[37] Galobardes B, Shaw M, Lawlor DA, Lynch JW, Davey SG (2006). Indicators of socioeconomic position (part 1). JEpidemiolCommunity Health.

[38] Duda C, Mahon I, Chen MH, Snyder B, Barr R, Chiles C (2011). Impact and costs of targeted recruitment of minorities to the National Lung Screening Trial. Clin Trials Lond Engl.

